# Evaluating the influence of service quality, hedonic, and utilitarian value on shopper's behavioral intentions in urban shopping malls during the COVID-19 pandemic

**DOI:** 10.1016/j.heliyon.2022.e12542

**Published:** 2022-12-20

**Authors:** Ardvin Kester S. Ong, Yogi Tri Prasetyo, Barbara Eliza Vallespin, Satria Fadil Persada, Reny Nadlifatin

**Affiliations:** a School of Industrial Engineering and Engineering Management, Mapua University, Manila, Philippines. 658 Muralla St., Intramuros, Manila, 1002, Philippines; b International Program in Engineering for Bachelor, Yuan Ze University, 135 Yuan-Tung Rd., Chung-Li, 32003, Taiwan; c Department of Industrial Engineering and Management, Yuan Ze University, 135 Yuan-Tung Rd., Chung-Li, 32003, Taiwan; d School of Graduate Studies, Mapúa University, Manila, Philippines. 658 Muralla St., Intramuros, Manila, 1002, Philippines; e Entrepreneurship Department, BINUS Business School Undergraduate Program, Bina Nusantara University, Jakarta, 11480, Indonesia; f Department of Information Systems, Institut Teknologi Sepuluh Nopember, Kampus ITS Sukolilo, Surabaya, 60111, Indonesia

**Keywords:** Shopping mall, Service quality, Shopper's satisfaction, SERVQUAL, Structural equation modeling

## Abstract

Shopping malls are subjected to a lot of changes during the COVID-19 pandemic, especially in the Philippines. The objective of the study was to evaluate the influence of service quality, hedonic, and utilitarian value of malls on shopping mall goers' behavioral intentions during the COVID-19 pandemic. SERVQUAL five dimensions were utilized to represent the service quality aspect, while utilitarian and hedonic values are used to embody the shopping mall values among Filipino citizens. Additionally, we included convenience and social experience as part of our model, to which relationships were assessed toward the utilitarian and hedonic shopping mall values, respectively. An online survey was distributed and collected 519 valid responses among Filipino shopping mall goers. Using Structural Equation Modeling (SEM), results showed that for service quality aspect, tangibles, empathy, and assurance had significant effects on shopper's shopping mall satisfaction during the COVID-19 pandemic. On the other hand, both shopping mall values present positive effects on shopper's satisfaction on which utilitarian value gave the strongest influence in the overall model, followed by the hedonic value. Convenience and social experience also showed positive effects on utilitarian and hedonic values, respectively. The utilized extended framework from SERVQUAL dimensions and hedonic and utilitarian values to measure satisfaction and behavioral intentions was seen to be applicable to measure human behavior and lifestyle studies. Overall, the satisfaction from both service quality and shopping mall values aspect strongly influences shoppers' behavioral intention in going to the mall.

## 1 Introduction

The Philippines has been acknowledged as a home of big shopping malls (Alegado &Yap, 2020). Most parts of the country have shopping malls and are still progressing despite the Philippines being a small country (Garcia, 2020). Garcia (2020) also stated that the Philippines has the biggest shopping mall in all Asian countries. According to the article by Federis (2008), eighty (80) percent of the Filipino population was recorded to visit shopping malls monthly.

Throughout the years, it was seen that there was increased growth in mallgoers. Among the entire sector in 2013, shopping malls in the Philippines contributed to 15% of the Gross National Product (GNP) (Tacujan, 2013). Soliman (2018) indicated that one of the largest and leading shopping mall operators in the Philippines earned 59 percent revenue in Philippine Peso (PHP), about 13.9 billion from its mall business from 2017 to 2018. Federis (2008) indicated that shopping became part of the Filipino lifestyle throughout the years. In addition, traditional malls in the Philippines remain to continuously operate and grow to adapt and co-exist with the thriving trends even with the emergence of online shopping and e-commerce (Soliman, 2018). Therefore, shopping malls and consumers’ satisfaction should be explored.

Recently, the COVID-19 pandemic had impacted the shopping malls in the country. The Business Today news reported a 50% decline in revenue among shopping malls (Kenge and Khan, 2020). However, shopping malls are still trying to break through the new normal by decreasing rent, trying to find ways to keep the market afloat, and catering to people's needs. Consumer and shopping mall typologies were indicated to highly change during the COVID-19 pandemic (Hartono et al., 2021). Phaosanthianphan and Leelasantitham (2020) explained that the behavior of consumers also changed due to the strict lockdown implementation. Hartono et al. (2021) provided an analysis of the difference in patterns of people when shopping during the COVID-19 pandemic.

The closing of malls across different countries due to the COVID-19 has affected the economy, jobs, and businesses (Pantano et al., 2020; Prasetyo et al., 2020). Pantano et al. (2020) elaborated that there was a 3.2% and 2.9% GDP decrease due to the closure of shopping malls in early and late 2020 for UK and US, respectively. To which, it was indicated that other countries also felt the decline due to new strategies and development for new normal living adapting to the COVID-19 pandemic. Ong et al. (2021c) elaborated on the change in buying behavior, pattern, and spending habits of people in the Philippines due to the COVID-19 pandemic; and has caused the Philippines' economic loss to around $13,000. The need for the evaluation of consumer behavior in relation to shopping should be reevaluated to enhance the continuance intention of shoppers and mall-goers. With that, different studies were considered with regard to shopping malls all around the world but were relatively specific; thus, the creation of strategies was limited and cannot be generalized.

Different studies have focused on how stores in the shopping mall are struggling during the COVID-19 pandemic to keep afloat. Hartono et al. (2021) showed how the segmentation of people in Indonesia highlighted adaptive behavioral patterns. Their results showed that people who are health conscious and of the younger generation would adopt online shopping. Similarly, other countries that are in line with health consciousness also applied the adoption to online shopping. However, physically going to shopping malls may sometimes be dependent on the cultural aspect. Van Eeden (2007) showed that geographical characteristics such as age, class, and race would affect behavioral intentions, especially cultural differences. Ong et al. (2021c) presented that despite the online shopping availability, people would tend to want the physical display and feel of the material before they purchase the product.

Nanda et al. (2021) considered urban management and how the pandemic accelerated the utilizing e-commerce and digitalization. Their results showed that there is a need for physical shops to reposition their businesses. Moreover, Pantano et al. (2020) did a commentary review regarding consumers and managers of retailers during the COVID-19 pandemic. They urged them to rethink and strategize a business plan during the COVID-19 pandemic. Kenge and Khan (2020) highlighted the sudden change in online shopping among consumers during the peak of COVID-19. This led to a great demise among shopping malls around the world; with a shift of 62% of consumers preferring online shopping. Therefore, this resulted in a decrease in the economy as the sudden change of living brought about by the COVID-19 pandemic affected everyone (Belmonte et al., 2022; Cahigas et al., 2022; Eger et al., 2021; Kusonwattana et al., 2022). This showed how the current pandemic greatly affected shopping malls, the people, and the economy in general as shopping malls brought great economic income to different countries (Soliman, 2018).

In addition, there were studies that explored shopping malls in different places throughout the years before the COVID-19 pandemic (Soliman, 2018; Federis, 2008; Krey et al., 2022; Kesari and Atulkar, 2016). However, these studies mostly focused on determining different factors that significantly affect the shopper's shopping mall satisfaction and loyalty (Krey et al., 2022). Kesari and Atulkar (2016) focused on utilitarian and hedonic values among shoppers and its possible impact on satisfaction. Their findings showed that both values significantly influenced shoppers on how they perceived their satisfaction towards the shopping mall. Moreover, different studies mainly focused on specific variables such as factors exclusive to shopper's shopping mall values (El-Adly and Eid, 2016), shopper's congruity and well-being (Kwon et al., 2016), or mall/store image and environment. These factors were then used to determine the relationship to shopper's mall satisfaction and loyalty. It could be seen that there is a scarcity of studies that mainly used the SERVQUAL model as variables for a possible significant effect to shopper's shopping mall satisfaction and behavioral intention during the COVID-19 pandemic.

SERVQUAL dimensions were indicated to be a benchmark for evaluating customer service and customer satisfaction established and promoted in a new environment (Chuenyindee et al., 2022). It was indicated that utilizing the dimensions as a model would create an understanding for the development and analysis of behavior among consumers, reshape strategies, and develop the business to cater to consumers (Chuenyindee et al., 2022). In addition, for further holistic measurement, the values of human behavior such as the utilitarian and hedonic values should be considered which affects consumer satisfaction (Magkopa, 2016). Since the change in business strategy, pattern, and activity are in place due to the COVID-19 pandemic, reassessment of their current strategies should be explored. Nanda et al. (2021) presented different challenges and business shifts among retail industries, in relation to this study – retail industries especially in shopping malls. The analysis of satisfaction among consumers and their intention to continue going to shopping malls despite the challenges and protocols implemented should be assessed for the new normal.

Considering details from previous studies mentioned, this paper intended to bridge the gap by studying shopping mall consumers' behavioral intentions and their perception of satisfaction, mainly in the Philippines. Relating to the study of Ong et al. (2021c), it was depicted that people in the Philippines would want to buy items that they see and feel physically as much as possible; thus, the difference in the available e-commerce where consumers lack the motivation to purchase items. In addition, the COVID-19 pandemic was mainly focused on due to the change in lifestyle and businesses following the strict protocol set by the government. This study intends to evaluate the shopping mall's value, quality of service, and the perception of customers during the COVID-19 pandemic. Extending the SERVQUAL model, this study included hedonic (HV) and utilitarian values (UV) to measure shoppers' satisfaction (SS) and behavioral intention (BI). The result of this study will contribute and provide analysis that could be utilized by different shopping malls around the world. Since the current trend is the application of e-commerce, Petcharat and Leelasantitham (2021) explained how user behavior and adoption have changed the usage behavior of people. Thus, there is an effect of actual and online purchases of people worldwide.

By understanding what contributes to the satisfaction of consumers' behavioral intention to go to shopping malls despite the COVID-19 pandemic and new protocols, the development of business strategies would help set guidelines that could be implemented in shopping malls around the world. Since the shopping malls could be generalized to be a place where food, activities, and purchases are made, it could be deduced that the findings of this study may be applicable to different countries – taking into consideration the cultural differences should be noted. Moreover, the result could be used as a decision-making tool and create strategies for customer satisfaction during the new normal. The satisfaction of customers would lead to an increase in mallgoers, leading to an increase in profit. The flow of the research is as follows: (1) Introduction which covers the problem and research gap, objective, and application of the study, (2) conceptual model framework to provide additional literature and model utilized in this study, (3) methodology, (4) results, (5) discussion and implications, and (6) conclusion of the study.

## 2 Conceptual model framework

**Figure 1:**
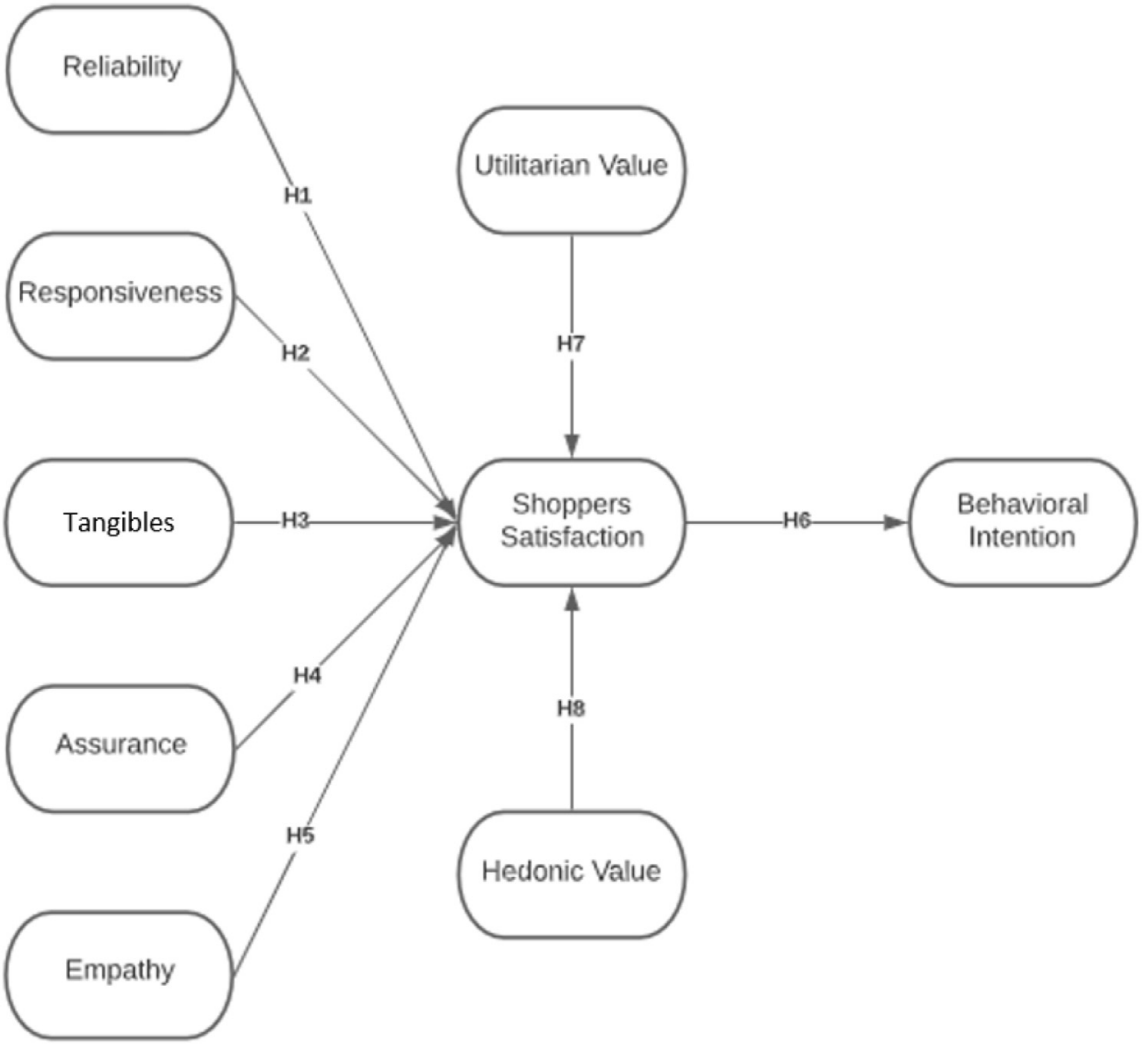
Conceptual framework.

The study extended the SERVQUAL model for the conceptual framework including hedonic and utilitarian value for the shopper's satisfaction, seen in Figure 1. Chuenyindee et al. (2022) posited how the SERVQUAL dimensions can measure marketing and business evaluation holistically. Especially in new environments and settings, the SERVQUAL dimensions was suggested to be utilized since this benchmarking framework creates a baseline for the re-evaluation of new service provided and their influence on customer satisfaction. However, behavioral aspects are said to be of value when personal factors are considered (Kesari and Atulkar, 2016). Their study concluded that these two factors influenced customer satisfaction. To which, this study intended to measure the positive behavioral intentions of shoppers from the different latent variables affecting satisfaction during the COVID-19 pandemic.

SERVQUAL may be utilized in the analysis of shoppers’ perception of the current service quality of shopping malls and how it affects SS during the new normal. Dzisi et al. (2021) showed that reliability and responsiveness were significant factors that are relevant to the mobility-as-a-service features to service. Tumsekcali et al. (2021) also utilized SERVQUAL dimensions in public service transport in Turkey showing that factors such as empathy and tangibles had a significant effect during the COVID-19 pandemic. Moreover, Valiyappurakkal (2021) utilized the SERVQUAL dimensions during the new normal and showed that the dimensions affected satisfaction.

Reliability is the performance of a service provider to provide services dependably, on time, and accurately. Lee et al. (2019) presented reliability as a latent variable that affects customer satisfaction which would affect the continuous and behavioral intentions of consumers. Shahin and Pourhamidi (2011) expounded on relating reliability to satisfaction for mall shoppers, explaining its direct significant relationship. In addition, Zhao et al. (2017) showed how the environment where shopping malls are affects individual differences. To which, it was posited that the reliability of service despite the differences greatly affected SS. With that, it was hypothesized that:

H1: Reliability positively and directly affects SS.

Responsiveness is explained as the effectiveness, promptness, and acknowledgment of service among consumers (Lee et al., 2019). Several studies justified the effect of responsiveness on customer SS (Tiglao et al., 2020). It was stated that the more responsive the service providers are to customer concerns, the more positive the influence is toward customer SS. Similarly, Chou et al. (2011) presented the direct and positive significant effect of responsiveness on SS in service quality. Thus, it was hypothesized that:

H2: Responsiveness positively and directly affects SS.

In relation to this study, tangibles are referred to the cleanliness and appearance of shopping malls. Similarly, Alam and Mondal (2019) showed how appearance and sanitation would affect the satisfaction of consumers. The service quality is greatly affected by tangibles (Mikhaylov et al., 2015). It was posited that the environment where a person greatly influences their perception of overall sanitation. Since the COVID-19 pandemic requires constant sanitation to reduce the spread of the virus, Chuenyindee et al. (2022) presented the direct and significant relationship of which to SS. Thus, it was hypothesized that:

H3: Tangibles positively and directly affects SS.

Assurance of service providers to cater to the needs of the customers is highly relevant in the retail industry (Tumsekcali et al., 2021). Studies such as that of Chuah and Hilmi (2011) and Sam et al. (2018) presented a direct significant relationship between assurance of service quality and customer satisfaction. Sam et al. (2018) explained that assurance is one of the highlighted factors that affect SS. The knowledge and courtesy of service providers would present a high-quality service being provided to consumers. This promotes trust and confidence among shoppers in the service obtained from shopping malls (Sam et al., 2018). Thus, it was hypothesized that:

H4: Assurance positively and directly affects SS.

The relationship between service providers is highly influenced by their way of relating and feeling for their customers (Chuah and Hilmi, 2011). Empathy as a latent variable depicts this emotional aspect. Alam and Mondal (2019) presented how empathy has the highest significant direct effect on SS. Chuenyindee et al. (2022) showed that when service providers show high empathy, customers would feel the importance which related to their satisfaction and behavioral intentions. Moreover, amidst the COVID-19 pandemic, empathizing with customers would promote high SS among shoppers (Tumsekcali et al., 2021). Thus, it was hypothesized that:

H5: Empathy positively and directly affects SS.

Hong et al. (2021) discussed how businesses have difficulty in attaining satisfactory levels among customers during the new normal, thus affecting customers’ behavioral intentions. Their study showed that when customers trust the service provided, the behavioral intentions of customers would be evident. Moreover, Kostromitina et al. (2021) showed that service, experience, and quality would have great satisfaction among customers and how it leads to positive behavioral intentions (Li et al., 2020). With high satisfaction, it was seen that it will lead to positive behavioral intentions among consumers (Du et al., 2020). In addition, higher satisfaction would lead to positive motivation which will encourage behavioral intentions (Jattamart and Leelasantitham, 2020). Thus, it was hypothesized that:

H6: SS positively and directly affects BI.

Utilitarian Value (UV) pertains to the consumer's reflection on the purchase and acquisition of products (Jones et al., 2006). This latent variable covers their convenience when it comes to purchasing. In addition, Jones (2006) indicated that Hedonic Value (HV) corroborates with the consumer's senses, emotions, and ideals when purchasing. This latent covers the consumers' social experience. To which, their paper presented the importance of both HV and UV in shopping and consumer purchase intention. Similarly, the study of Babin et al. (2005) showed the strong relationship between UV and HV to SS – leading to a perception of in the importance of integrating both values in retail experiences. Thus, Jones et al. (2006) deciphered the importance, relationship, and differences between both values when it comes to retail industries and shopping malls.

Makgopa (2016) stated that shopping malls have not just become a place of purchase for shoppers' needs but also a space for socialization. Both values, Kesari and Atulkar (2016) considered both UV and HV in assessing satisfaction among mall shoppers. It was seen that the lifestyle of consumers affected their values which leads to a positive SS. In addition, if the values are effectively delivered, a positive direct effect on SS would be evident. Similarly, To et al. (2007) also presented the importance of shopping values in the e-commerce setting. UV and HV would greatly affect SS which would lead to a positive behavioral intention among customers. Lastly, Allard et al. (2009) explained that UV is highly effective for customer orientation with the evaluation of shopping malls, followed by HV. Therefore, it was hypothesized that:

H7: UV positively and directly affects SS.

H8: HV positively and directly affects SS.

## 3 Methodology

### 3.1 Population and sample

This study measured the behavioral intentions of consumers to go to shopping malls during the COVID-19 pandemic by collecting a total of 519 respondents via convenience sampling through an online survey. Through convenience sampling, the online survey was distributed through several social media platforms due to the social distancing implemented in the country similar to the study of Ong et al. (2021a). Moreover, the liberty to exit the survey was available and their data would not be collected. Only those who completed the survey were recorded in the system database. Following the suggestion of Hair (2010), 500 respondents would suffice to generalize the findings of a model with 8 or more latent variables. This study considered respondents who have gone to the malls and are residing in the Philippines during the COVID-19 pandemic. This study was approved by Mapua University Research Ethics Committees (FM-RC-20-52). An informed consent was obtained from all participants prior to the data collection.

### 3.2 Participants

Presented in Table 1 are the descriptive statistics of the demographics. The respondents were 63.20% female and 34.68% male, the majority between 15-44 years old. The respondents were mostly unemployed and students (55.1%%) and employed (43.35%) who are single (83.62%). Most of the respondents have a shopping budget of less than 2,000 PHP to 5,000 PHP ($35-$88) per month (80.92%) who goes to the shopping mall either once or twice a month (70.13%).

**Table 1:** Descriptive analysis of demographics (*n = 519*).

Characteristics	Category	N	%
Gender	Male	180	34.68%
	Female	328	63.20%
	Other	11	2.12%
Age	Less than 15 years old	2	0.39%
	15–24 years old	278	53.56%
	25–34 years old	162	31.21%
	35–44 years old	60	11.56%
	45–54 years old	7	1.35%
	more than 54 years old	10	1.93%
Occupation	Student	255	49.13%
	Unemployed	31	5.97%
	Employed	225	43.35%
	Retired	8	1.54%
Marital Status	Single	434	83.62%
	Married	84	16.18%
	Divorced	1	0.19%
Shopping budget per month	Less than PHP 2,000.00 (<$35)	266	51.25%
	PHP 2,000.00 - PHP 5,000.00 ($35-$88)	154	29.67%
	PHP 5,001.00 - PHP 8,000.00 ($88-$140)	46	8.86%
	PHP 8,0001.00–11,000.00 ($140-$193)	22	4.24%
	More than PHP 11,000.00 (>$193)	31	5.97%
Frequency of visits to shopping mall per month	Daily	4	0.77%
	Weekly	72	13.87%
	Thrice a month	79	15.22%
	Twice a month	136	26.20%
	Once a month	228	43.93%
Number of Companions when visiting the shopping mall for the past months during Covid-19	Solo	86	16.57%
	With 1–2 people	312	60.12%
	With 3–5 people	53	10.21%
	More than 5 people	9	1.73%
	Solo, With 1–2 people	40	7.71%
	Solo, With 1–2 people, With 3–5 people	4	0.77%
	Solo, With 3–5 people	2	0.39%
	With 1–2 people, More than 5 people	1	0.19%
	With 1–2 people, With 3–5 people	11	2.12%
	With 3–5 people, More than 5 people	1	0.19%

To elaborate more on the descriptive statistics, a cross-tabulation analysis was conducted as presented in Table 2. The table was created to identify the relationship between the different demographic characteristics on the frequency of visiting shopping malls per month during the COVID-19 pandemic. It could be seen that single females of 15–24 or 25–34 years old who are either students or employed would most likely visit shopping malls even during the COVID-19 pandemic. These criteria were established even in early 2006 when this behavior demonstrates identity formation and social spatialization which females have sustained throughout the years (van Eeden, 2007).

**Table 2:** Cross-tabulation analysis.

Factor	Characteristics	Frequency					Total
		Daily	Weekly	Thrice a month	Twice a month	Once a month	
Gender	Male	2	30	28	46	74	180
	Female	1	41	49	89	148	328
	Other	1	1	2	1	6	11
	% distribution	0.008	0.139	0.152	0.262	0.439	519
Age	Less than 15	0	0	1	0	1	2
	15–24 years old	3	21	45	62	147	278
	25–34 years old	1	35	25	40	61	162
	35–44 years old	0	13	7	24	16	60
	45–54 years old	0	1	0	5	1	7
	More than 54 years old	0	2	1	5	2	10
	% distribution	0.008	0.139	0.152	0.262	0.439	519
Occupation	Student	3	19	41	51	141	255
	Unemployed	0	9	4	9	9	31
	Employed	1	42	33	73	76	225
	Retired	0	2	1	3	2	8
	% distribution	0.008	0.139	0.152	0.262	0.439	519
Marital Status	Single	3	52	67	105	207	434
	Married	1	20	12	30	21	84
	Divorced	0	0	0	1	0	1
	% distribution	0.008	0.139	0.152	0.262	0.439	519
Shopping Budget	Less than PHP 2,000.00	1	14	41	50	160	266
	PHP 2,000.00 - PHP 5,000.00	1	29	24	54	46	154
	PHP 5,001.00 - PHP 8,000.00	1	11	6	16	12	46
	PHP 8,0001.00–11,000.00	0	9	5	5	3	22
	More than PHP 11,000.00	1	9	3	11	7	31
	% distribution	0.008	0.139	0.152	0.262	0.439	519
Companions	Solo	1	10	10	21	44	86
	With 1–2 people	3	55	45	89	120	312
	With 3–5 people	0	2	10	9	32	53
	More than 5 people	0	1	3	1	4	9
	Solo, With 1–2 people	0	3	6	12	19	40
	Solo, With 1–2 people, With 3–5 people	0	0	2	1	1	4
	Solo, With 3–5 people	0	0	1	0	1	2
	With 1–2 people, More than 5 people	0	0	0	0	1	1
	With 1–2 people, With 3–5 people	0	1	2	2	6	11
	With 3–5 people, More than 5 people	0	0	0	1	0	1
	% distribution	0.008	0.139	0.152	0.262	0.439	519

Van Eeden (2007) explained how the criteria of demographics present a dominant ideology in consumer capitalism which centered around class, gender, space, and race; which established a social practice among women. To which, a budget of 2,000PhP ($35) or less was considered with approximately 1–2 people going with a person for shopping mall visitation. With the COVID-19 pandemic affecting the economy of countries, especially in the Philippines, De Castro et al. (2020) justified how having a low budget during the COVID-19 became substantial for commodities and other necessities during the pandemic. This, proper budgeting while still being to achieve a level of leisure was seen in this study's demographic characteristics.

### 3.3 Questionnaire

Table 3 presents the measures that were adapted from different studies which were utilized in this study. A 7-point Likert scale survey focused on measures for the variables covering reliability, responsiveness, empathy, assurance, and tangibles for the SERVQUAL model (Parasuraman, 1991). Moreover, the variables such as hedonic and utilitarian value, satisfaction, and behavioral intention were also measured by applying the studies of El-Adly and Eid (2016) and Kesari and Atulkar (2016).

**Table 3:** The construct and measurement items.

Variable	Code	Constructs	Reference
Reliability (R)	R1	Shopping malls have the products I need even during COVID-19	Parasuraman et al. (1991; Cronbach's alpha = 0.831)
	R2	Shopping malls have services I need even during COVID-19	
	R3	Shopping malls customer service during COVID-19 is still reliable	
	R4	Shopping malls and stores open and close on time during COVID-19	
	R5	Shopping malls staff (e.g., guards, sales lady/man) deliver their tasks correctly during COVID-19.	
Assurance (A)	A1	Shopping malls instill confidence to every shopper that it follows every standard COVID-19 safety protocols and sanitization mandated by the Government.	Parasuraman et al. (1991; Cronbach's alpha = 0.760)
	A2	I feel safe going to shopping malls with its current safety and sanitization protocols.	
	A3	Shoppers get adequate support from Shopping mall management and staff in terms of protection to COVID-19.	
	A4	Shopping mall management and staff during COVID-19 is trustworthy.	
Tangibles (T)	T1	Shopping malls look clean and sanitized especially during COVID-19.	Parasuraman et al. (1991; Cronbach's alpha = 0.636)
	T2	Shopping mall staff are always seen clean and wearing mask and/or face shield.	
	T3	Shopping malls overall interior and decors look visually appealing even during COVID-19.	
	T4	Shopping malls have posted COVID-19 preventive guidelines and protocols that are visible for shoppers inside the premises (e.g., pathways guide where to walk in observance of social distancing, protocols printed graphics can be easily understand prior going in a store).	
Empathy (E)	E1	Shopping malls have the shopper's interest at heart.	Parasuraman et al. (1991; Cronbach's alpha = 0.755)
	E2	Shopping malls operating hours and days are convenient to shoppers especially during COVID-19.	
	E3	Shopping malls and stores understand the specific needs of their customers.	
	E4	I feel prioritized by the customer service and/or shopping mall staff whenever I asked for assistance or have complaints.	
	E5	Shopping malls staff communicate to shoppers respectfully and clearly.	
Responsiveness (RS)	RS1	Shopping malls provide timely information to shoppers of changes in its operation days/hours and protocols during COVID-19	Parasuraman et al. (1991; Cronbach's alpha = 0.694)
	RS2	Shopping malls staff can quickly answer shopper's query and confusion especially on the protocols during COVID-19.	
	RS3	Shopping malls staff have initiative in ensuring and maintaining discipline and organization inside the mall.	
	RS4	Despite the strict protocols during COVID-19, shopping malls and store staff try their best in lessening waiting time/queue of shoppers.	
Hedonic Value (HV)	H1	I don't mind getting less interaction with mall staff (i.e., salesmen/saleslady) in shopping malls as one of the safety protocols during COVID-19.	Kesar and Atulkar (2016; Cronbach's alpha = 0.790) El-Adly and Eid (2016; Cronbach's alpha = 0.760) El-Adly and Eid (2016; Cronbach's alpha = 0.770) Cronbach's alpha = 0.770 Cronbach's alpha = 0.810 Cronbach's alpha = 0.770
	H2	I still find it comfortable going to shopping malls with a companion even during COVID-19.	
	H3	I find it safe to meet-up with friends and/or family over shopping or lunch/dinner in shopping malls even during COVID-19.	
	H4	I find it enjoyable spending time with friends and/or family in shopping malls even during COVID-19.	
	H5	I still able to meet new people in shopping malls even during COVID-19.	
	SE1	I don't mind getting less interaction with mall staff (i.e., salesmen/saleslady) in shopping malls as one of the safety protocols during COVID-19.	
	SE2	I still find it comfortable going to shopping malls with a companion even during COVID-19.	
	SE3	I find it safe to meet-up with friends and/or family over shopping or lunch/dinner in shopping malls even during COVID-19.	
	SE4	I find it enjoyable spending time with friends and/or family in shopping malls even during COVID-19.	
	SE5	I still able to meet new people in shopping malls even during COVID-19.	
Utilitarian Value (UV)	U1	I plan ahead to make my shopping trip valuable.	El-Adly and Eid (2016; Cronbach's alpha = 0.790) Kesari and Atulkar (2016; Cronbach's alpha = 0.710)
	U2	I usually go to shopping malls to just buy certain things.	
	U3	Once I accomplish the task that I need to do inside the shopping mall I directly go home.	
	U4	Shopping malls are a good place to shop effortlessly.	
	U5	Despite COVID-19, shopping directly at the mall makes me perceive more utility and value for the purchase.	
	C1	I visit the shopping mall because of its convenient location.	
	C2	I go to shopping malls only because of the convenience of having variety of product offerings in one roof.	
	C3	I find it great and convenient how spacious the mall grounds and stores nowadays when I go to the shopping mall.	
	C4	I still find it easy to access shopping malls in spite of the added protocols during COVID-19.	
	C5	A good shopping experience is one that is over very quickly.	
Shopping Satisfaction (SS)	SS1	I am satisfied on the safety protocols implemented by shopping malls during COVID-19	Kesari and Atulkar (2016; Cronbach's alpha = 0.730)
	SS2	I am satisfied on how clean shopping malls appear whenever I visit during Covid-19	
	SS3	I am satisfied with the product and service quality I get in shopping malls during COVID-19	
	SS4	I am satisfied on how shopping malls staff operate during COVID-19	
	SS5	I am satisfied with my decision on going to shopping malls even during COVID-19	
Behavioral Intentions (BI)	BI1	I will continue going to shopping malls during COVID-19 and in the future.	Prasetyo et al. (2021; Cronbach's alpha = 0.782)
	BI2	I will continue to get products and services in shopping malls during COVID-19 and in the future.	
	BI3	I am willing to encourage and recommend to my friends and family going to shopping malls during COVID-19 and in the future.	
	BI4	I can see myself increase my visit to shopping malls to shop going forward.	
	BI5	I can see myself increase my visit to shopping malls to have fun going forward.	

Under the SERVQUAL model, twenty-two (22) adapted items were allocated among the five dimensions (Parasuraman et al., 1988, 1991), while the rest of the constructs were adapted from other related studies, measured using SEM. SEM is an advanced statistical technique that is utilized to measure the causal relationship between the latent variable and constructs (Antwi-Afari et al., 2021; Ong et al., 2021b; Ong et al., 2021a).

## 4 Results

**Figure 2:**
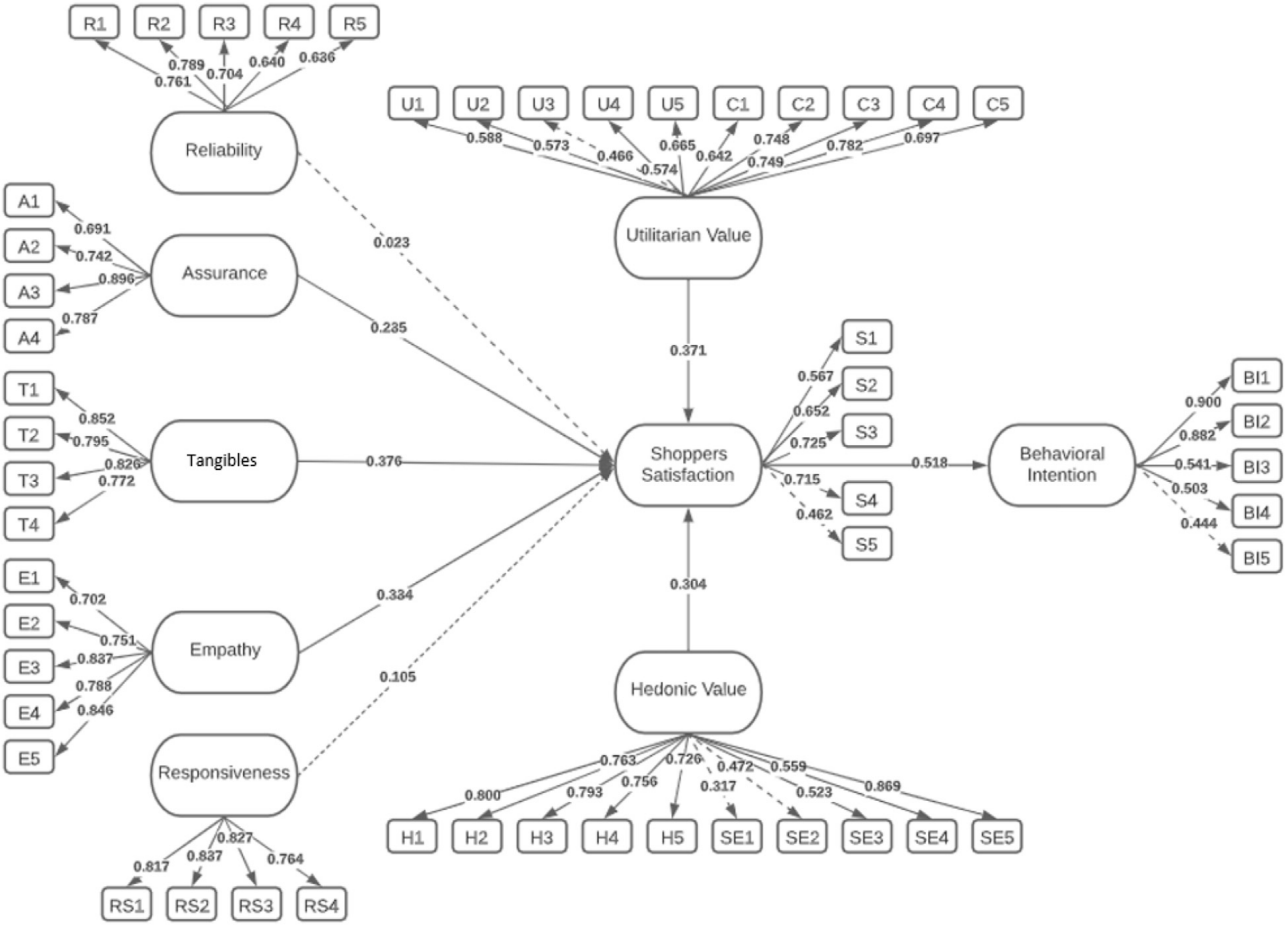
Initial SEM for evaluating shopping mall values and service quality.

**Figure 3:**
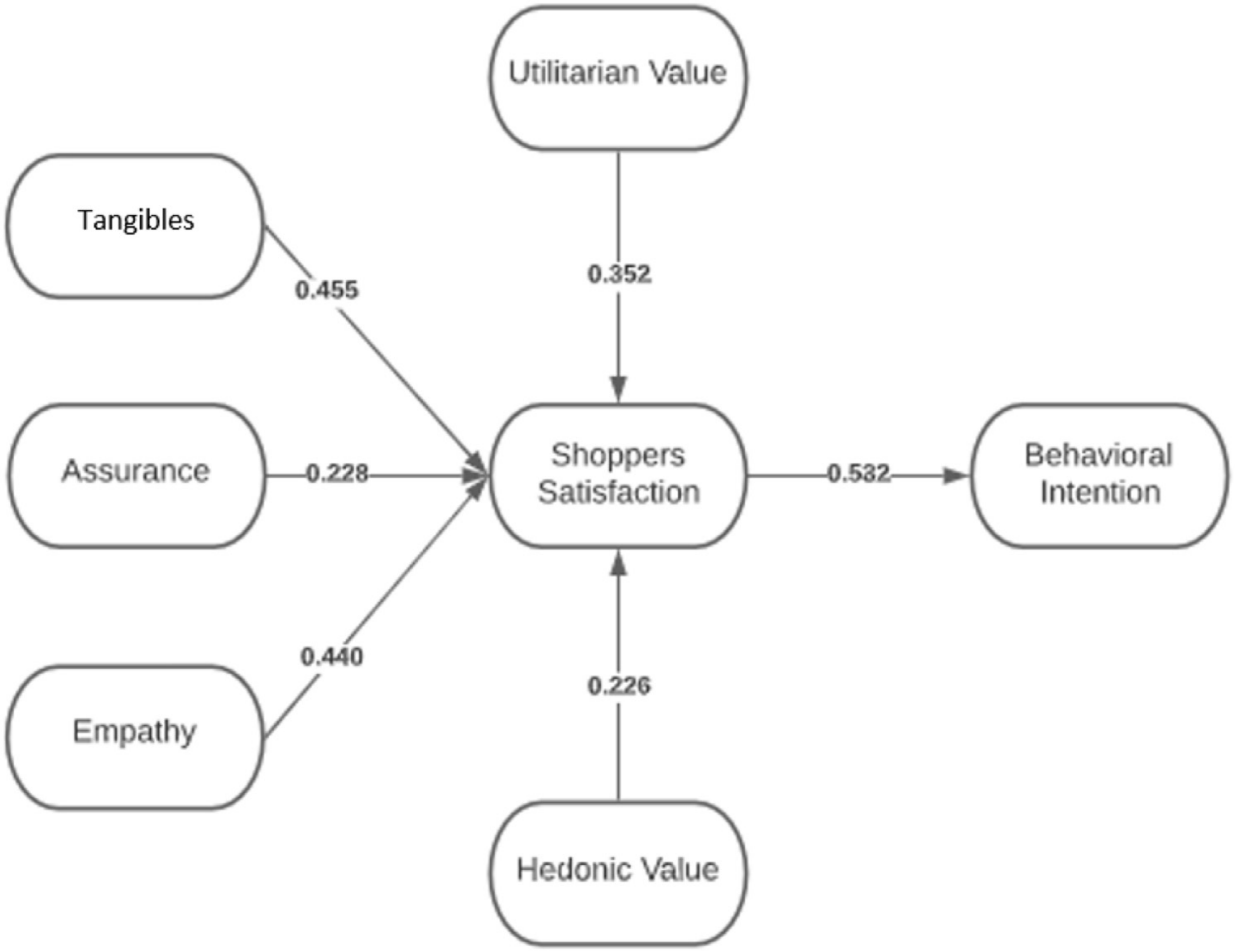
Final SEM for evaluating shopping mall values and service quality.

The initial model seen in Figure 2 is for evaluating shopping mall values by extending the SERVQUAL model. Running the Shapiro-Wilks test showed a value within the threshold, ±1.96. This presents that the collected data could be utilized without bias and is normal. On the other hand, following the suggestion of Hair (2010), insignificant latent variables (p-value >0.05) and factor loading (<0.50) could be removed to enhance the model fit (Ong et al., 2021a). To which, hypotheses 1 and 2 were removed since the p-value were greater than 0.05. Figure 3 represents the final model utilized for SEM analysis.

Checking the model fit, Table 4 shows that all measures are within the suggested threshold by Gefen et al. (2000) and Steiger (2007). This indicates that the model is acceptable (Hair, 2010; Gefen et al., 2000; Steiger, 2007). Utilizing the Harman's Single Factor test, no common method bias was present with a total variance value of 23.62%. It was stated in the study of Podsakoff et al. (2003) that a common method bias threshold would not be present if the total variance has less than 50% value.

**Table 4:** Model fit estimates and suggested measure of acceptance.

Goodness of fit measures of the SEM	Parameter Estimates	Minimum cut-off	Recommended by
Goodness of Fit Index (GFI)	0.806	>0.80	Gefen et al. (2000)
Adjusted Goodness of Fit Index (AGFI)	0.828	>0.80	Gefen et al. (2000)
Root Mean Square Error of Approximation (RMSEA)	0.066	<0.07	Steiger (2007)
Incremental Fit Index (IFI)	0.852	>0.80	Gefen et al. (2000)
Tucker Lewis Index (TLI)	0.833	>0.80	Gefen et al. (2000)
Comparative Fit Index (CFI)	0.851	>0.80	Gefen et al. (2000)

Average Variance Extracted (AVE), Composite Reliability (CR), and Cronbach's alpha (α) seen in Table 5 presents the reliability and validity of the constructs. Hair (2010) suggested that these values should be greater than 0.7 with AVE greater than 0.5 to indicate the internal validity and reliability of the constructs.

**Table 5:** Reliability and Validity of constructs.

Latent Variables	Items	Cronbach's α	Factor Loadings	Average Variance Extracted (AVE)	Composite Reliability (CR)
A	A1	0.932	0.691	0.612	0.862
	A2		0.741		
	A3		0.896		
	A4		0.787		
T	T1	0.945	0.825	0.648	0.880
	T2		0.763		
	T3		0.843		
	T4		0.787		
E	E1	0.948	0.701	0.620	0.890
	E2		0.750		
	E3		0.839		
	E4		0.792		
	E5		0.846		
HV	H1 H2	0.938	0.694 0.538	0.521	0.892
	H3		0.546		
	H4		0.564		
	H5		0.550		
	SE3		0.923		
	SE4 SE5		0.911 0.894		
UV	U1	0.947	0.703	0.508	0.902
	U2		0.733		
	U4		0.712		
	U5		0.647		
	C1		0.636		
	C2		0.737		
	C3		0.765		
	C4		0.785		
	C5		0.681		
S	SS1	0.958	0.669	0.517	0.810
	SS2		0.766		
	SS3		0.728		
	SS4		0.710		
BI	BI1	0.932	0.916	0.676	0.891
	BI2		0.918		
	BI3		0.783		
	BI4		0.639		

To further assess the internal validity of the constructs utilized, Hair (2010) suggested utilizing the Fornell-Larcker Criterion (FLC) and the Heterotrait-Monotrait (HTMT) Ratio. Presented in Table 6 are the results of the FLC which shows that the diagonal values are higher than the vertical values and their respective horizontal values. Gumasing et al. (2022) indicated that when the diagonal values are consistently higher than the corresponding horizontal and vertical values, internal validity has been achieved.

**Table 6:** Fornell-Larcker criterion.

	A	T	E	HV	UV	SS	BI
A	**0.782**						
T	0.726	**0.805**					
E	0.708	0.743	**0.788**				
HV	0.690	0.706	0.680	**0.722**			
UV	0.620	0.600	0.676	0.709	**0.713**		
SS	0.691	0.625	0.711	0.701	0.699	**0.719**	
BI	0.589	0.539	0.532	0.655	0.637	0.641	**0.822**

In addition, the HTMT ratio was conducted as presented in Table 7. German et al. (2022) indicated that a value less than 0.85 among all results would determine the model's acceptability and internal validity. Based on the results, the highest obtained value is 0.848 for one relationship. The rest of the values are lower than the threshold as well which presents the acceptability and validity of the model utilized in this study.

**Table 7:** Heterotrait-Monotrait ratio.

	A	T	E	HV	UV	SS
T	0.797					
E	0.807	0.791				
HV	0.793	0.793	0.748			
UV	0.848	0.805	0.802	0.777		
SS	0.800	0.799	0.787	0.797	0.839	
BI	0.679	0.617	0.609	0.830	0.700	0.676

Lastly, Table 8 presents the path analysis considering the standardized causal effects. The path analysis showed that SS to BI has the highest significant direct effect (β = 0.532; P = 0.016). Under SS, the highest significant direct effect is tangibles (β = 0.455; P = 0.006), followed by empathy (β = 0.440; P = 0.007), UV (β = 0.352; P = 0.005), assurance (β = 0.228; P = 0.005), and HV (β = 0.226; P = 0.004). In accordance, the indirect effect showed that tangibles, empathy, followed by UV, A, and HV were significant on behavioral intentions.

**Table 8:** Direct, indirect, and total effects.

No	Effect	Direct Effect	P-Value	Indirect Effect	P-Value	Total Effect	P-Value
1	HV→SS	0.226	0.004	-	-	0.226	0.004
2	E→SS	0.440	0.007	-	-	0.440	0.007
3	A→SS	0.228	0.005	-	-	0.228	0.005
4	T→SS	0.455	0.006	-	-	0.455	0.006
5	UV→SS	0.352	0.005	-	-	0.352	0.005
6	SS→BI	0.532	0.016	-	-	0.532	0.016
7	HV→BI	-	-	0.120	0.007	0.120	0.007
8	E→ BI	-	-	0.234	0.010	0.234	0.010
9	A→ BI	-	-	0.121	0.007	0.121	0.007
10	T→BI	-	-	0.242	0.008	0.242	0.008
11	UV→ BI	-	-	0.188	0.007	0.188	0.007

In addition, Table 8 presents the indirect effects, showing that the highest indirect effect is tangibles to SS (β = 0.242; P = 0.008), followed by empathy (β = 0.234; P = 0.010). The third highest indirect significant effect is seen on UV to BI (β = 0.188; P = 0.007). Lastly, the indirect significant effect of HV (β = 0.120; P = 0.007) and assurance (β = 0.121; P = 0.007) to BI is seen to be the same.

## 5 Discussion

This study aimed to determine whether shopping mall values and service quality during the COVID-19 pandemic affect the SS and BI toward the Philippines’ shopping malls. The result of the data and evaluation of the constructs are scrutinized in this section to understand and confirm the objectives and hypotheses of this study. Presented in Table 9 are the summarized accepted and rejected hypotheses.

**Table 9:** Hypotheses summary result.

Hypothesis	Relationship	Decision
1	Reliability → Shopper Satisfaction	Reject (p-value >0.05)
2	Responsiveness → Shopper Satisfaction	Reject (p-value >0.05)
3	Tangibles → Shopper Satisfaction	Accepted
4	Assurance → Shopper Satisfaction	Accepted
5	Empathy → Shopper Satisfaction	Accepted
6	Shopper Satisfaction → Behavioral Intention	Accepted
7	Hedonic Value → Shopper Satisfaction	Accepted
8	Hedonic Value → Shopper Satisfaction	Accepted

Reliability and Responsiveness were seen to be insignificant factors affecting SS. Following the study of Munusamy et al. (2010), responsiveness pertains to the sales agent's ability to provide agile response and good service to clients. Compared to the sales agents in the Philippines, the responsibilities of the sales representative (i.e. department stores) are to follow the customer around, know the whereabouts of items, and the location of products prior to being assigned to the floor. With that, it is evident that sales agents already have these characteristics upon being designated tasks or being responsible with customers. Thus, consumers are accustomed to the ability of sales agents to have high responsiveness – which has turned into the norm. Consequently, with the knowledge and training prior to being assigned, sales agents are reliable when it comes to customer needs and wants. Therefore, the norm has been established which is the reason why these two variables are deemed insignificant.

For the demographics illustrated in Table 1, female was seen to be the dominant respondent. In comparison to other genders, Pan and Zinkhan (2006) stated that women are shown to be more frequent shoppers. For a monthly shopping budget, most of the respondents answered that they allocated less than Php 2,000.00 and that their frequent visit to the mall during the COVID-19 pandemic is typically once a month. This is not surprising because there have been strict and additional protocols implemented, not just in shopping malls, but also in the Philippines as a whole. We can also key in the recession that has happened in the Philippines as a factor that may have affected the spending habit of shoppers. In addition, younger generations were seen to have a continuous intention to go to shopping malls. HV and UV were seen to be significant factors that influence the younger generation to have continuous usage behavioral intention during the COVID-19 pandemic (Pop et al., 2022).

Although it has been encouraged through protocols set in the Philippines that people should avoid social interaction, which includes going out with a companion or a group, shoppers still prefer 1 or 2 companions when shopping. This may be a reason for added motivation, influence on shopping assessment, and/or provided physical assistance by companions (Mora and Gonzáles, 2016). Similarly, the feeling of having companions to share excitement, fun, and happiness highlights satisfaction among mallgoers (Kesari and Atulkar, 2016). Comparing their study to the current findings, it could be posited that similar expressions of satisfaction are seen but of limited contact with others due to the COVID-19 pandemic.

For the Tangibles variable, constructs were significantly focused more on the cleanliness and protocol-based guidelines being promoted in the shopping malls. Moreover, the people and personnel followed the COVID-19 pandemic protocol guidelines. El-Adly and Eid (2016) supports that pleasing and comfortable environments are an important influence in the patronage of customers to shopping malls. The environment of shopping malls still has a crucial role during the new normal. Shoppers still expect and perceive shopping malls to be, not just clean and well-sanitized, but also visually appealing. Moreover, Tumsekcali et al. (2021) showed that both empathy and tangibles factors are significant when it comes to customer satisfaction during the new normal. Following the study of Uzir et al. (2021), this built trust among customers and would lead to SS. This reinforces the findings that shopping mall employees and the management, when seen diligently following safety protocols contribute to good service quality. Compared to other studies (Barabino et al., 2012; Chuenyindee et al., 2022), Tangibles as a latent variable was seen to be insignificant. It was deduced how people perceive their expectations to be different, depending on the setting. If people know that the quality of the environment or service is low, then Tangibles would not be considered significant.

Empathy is second in effect on SS. Shoppers deemed it as a highly positive indicator of quality service when there is clear and respectful communication from mall staff during COVID-19. Similar to the results of Uzir et al. (2021) and Valiyappurakkal (2021), empathy among service providers would lead to satisfaction. People going outside risk themselves to accommodate their personal needs and thus, their safety should be empathized by service providers. Moreover, understanding the specific needs of customers during the pandemic also resulted in one of the highest indicators of empathy. Shoppers will focus more on purchasing specific needs, like medicines or food, and less on electronic and other non-essential items. At the same time, shoppers find it also important that the mall's operational hours are conveniently accessible to everyone considering the curfews implemented in the country. The result of this study implies that if the Philippines' shopping malls can easily identify what the necessities are in terms of products and services to customers will affect SS toward shopping malls. In contrast, both studies by Mikhaylova et al. (2015) and Chuenyindee et al. (2022) presented no significance of Empathy as a latent variable towards service quality. It was deduced that when the service provided is open for public and is equally distributed to everyone, the individual service caterer should present attention towards the consumer to have a relative significant impact.

Lastly, the result of the quality of service relating to assurance also has a positive relationship with SS, wherein its highest indicator is shown as the sense of trustworthiness that shoppers feel about the shopping mall management and staff. Similar to the findings of Kliestik et al. (2022b), trust among consumers could be built to increase SS. In a similar way, their study showed the relationship between brand recognition and the continuous intention of consumers for purchasing and engagement. Shopping malls are expected to also give attention to building the trust and confidence of shoppers with their service, but also in finding better ways on protecting and preventing the spread and infection of the COVID-19 virus. This is in line with the next highest factor in our findings, wherein shoppers expect adequate support from mall staff and management in terms of protection against COVID-19. Valiyappurakkal (2021) also indicated that the SERVQUAL factors are inclined to the satisfaction of the consumers for service providers during the new normal, but is contrasted by the study of Mikhaylova et al. (2015). Thus, to increase satisfaction felt by shoppers in shopping malls, management must also investigate delivering assurance to shoppers.

Both UV and HV showed significant effects on SS; aligned with Allard et al. (2009). However, the inclination of shoppers’ values depends on their motivation or orientation. In this study, UV displayed a greater significance to SS versus HV during COVID-19. It is observable that most shoppers find more value and utility in going directly to shopping malls. In addition, the promotion of UV through the availability of everything consumers need under one roof has been established. It could be posited that consumer experience and convenience were key indicators of both HV and UV, respectively (Jones et al., 2006). This is in line with the results of Kesari and Atulkar (2016) and Khare (2011). The said value may come from monetary (e.g., discounts, on-sale items), a selection of a variety of products, and effortless shopping of getting what you need in one place (Arizzi et al., 2020; Kesari and Atulkar 2016). The results indicated that shoppers find that going to the mall nowadays is like a functional task that needs to be accomplished and that a sense of achievement afterward will then trigger their satisfaction. However, compared to online shopping, consumers are not able to have a sense of physical feel and actually see the products which is one reason why consumers would still want to go to shopping malls (Ong et al., 2021c). In addition, this has been supported by Phaosanthianphan and Leelasantitham (2020) and Frajtova Michalikova et al. (2022) on how behavioral change has been seen due to the COVID-19 pandemic, and that consumers are more likely to have differences in actual shopping and mobile shopping (Petcharat and Leelasantitham, 2021). In contrast, Zvarikova et al. (2022) depicted that when consumers would have decreased risk perception, an increase in the use of mobile applications would be seen.

Most shoppers identified HV in shopping malls, even nowadays, as a refreshing getaway. Similar to the study of Vinerean et al. (2022), HV in their study was seen to be a strong predictor of behavioral intention and satisfaction among tech-savvy users for m-commerce applications. However, the HV that shoppers experienced in shopping malls is more like an emotional experience and was found from previous studies to be greatly related to satisfaction (Arizzi et al., 2020). Currently, the Philippines is still under extended lockdown and continuously builds up negative emotions which impact greatly the mental health of people. From the study by Han et al. (2021), they suggested that these negative emotions should be mitigated by reducing emotional distress. Arizzi et al. (2020) discussed how the values are drivers of satisfaction for satisfaction in a business model during the COVID-19 pandemic. Thus, shopping malls may capitalize on this by providing entertainment, great visual interiors, and appealing marketing strategies to promote satisfaction and BI. It was discussed also by Babin and Krey (2020) that even if people do not buy anything (HV) during the shopping trip, and even if nothing was significant about the shopping trip (UV), SS may still be achieved during the new normal as long as they feel enjoyment.

It is on a positive note that the SS derived from the variables of service quality and shopping mall values showed a significant influence on BI. Despite the current situation of COVID-19, shoppers in the Philippines perceive themselves to continuously visit the mall, similar to the results of Kostromitina et al. (2021), Du et al. (2020), and Li et al. (2020). This is a good indicator that even with the struggles in the overall Philippines economy and even during the success of online shopping at present, people will still intend to go and shop in the malls. In fact, the result showed that shoppers foresee increasing their visits and shopping activities to malls going forward, even to the point of recommending to peers to go to shopping malls during the pandemic period.

Hong et al. (2021) had studied the BI of people during the COVID-19 pandemic. Their study presented that service providers had difficulties providing satisfaction during this unprecedented time. This has affected both consumers and retailers in shopping malls since they need mallgoers to continuously promote and engage in their activities and businesses. The relationship of service quality to satisfaction was seen to affect engagement and intention to continuously use services during the COVID-19 since people were seen to be afraid of the virus spreading and being infected (Chuenyindee et al., 2022). Thus, to continuously entice shoppers’ behavior in visiting a shopping mall, the management can use the data findings in this study to evaluate and work on their performance. Coinciding with the results of Alaimo et al. (2021), the satisfaction of customers should be of main importance among service providers as it is directly proportional to the sales of the company.

### 5.1 Theoretical implication

From the extended SERVQUAL dimension to measure service quality, it was seen that both utilitarian and hedonic values were directly significant which also influenced behavioral intentions. It was deduced that SERVQUAL dimensions alone may be utilized to analyze service quality and satisfaction, relating to the effect among behavioral intentions of consumers. However, the addition of values would strengthen the built relationship especially when lifestyle and emotions are involved. Similar to the study of Kesari and Atulkar (2016), the convenience, value for money, and products would greatly affect UV; while entertainment, exploration, happiness, and social status affected HV. All of these presented an indirect effect on behavioral intentions among shoppers to continuously go to shopping malls despite the COVID-19 pandemic. Thus, when evaluating satisfaction and behavioral intentions of consumers dealing with lifestyle, SERQUAL dimensions with extended values (*i.e.* hedonic and utilitarian) would develop a framework that holistically measures human behavior, similar to the findings of this study. Posited in this study is the application of the built framework for other human behavior-related studies that affect satisfaction and behavioral intentions.

### 5.2 Practical implication

The overall results of the study helped us derive the following practical applications from the service quality aspect that tackles the significant relationship of tangibles, empathy, and assurance to shoppers' shopping mall satisfaction. Relating to the study of Chuenyindee et al. (2022), the analysis using the SERVQUAL 5 dimensions would be essential in determining factors affecting consumer behavior in a relatively new setting. First, we suggest that mall management should focus on the quality of cleanliness of the interior and exterior of the mall, as well as the staff's personal hygiene. Compared to before the COVID-19 pandemic, consumers in the current era have taken into consideration safety protocols and the way this can be achieved is through the presentation of a clean environment – indicating that the area is clean and safe from virus spread. Second, shoppers expect malls to provide better communication thus, mall management should also look into making sure that announcements and messages are conveyed to shoppers clearly, timely, and accurately - malls could utilize social media (e.g., Facebook, Instagram, Twitter) in disseminating information and messages. This strategy could also be used for the malls to promote and interact with shoppers.

### 5.3 Managerial insights

In terms of shopping mall values, the study findings have revealed that the strongest relationship in the model is between UV to SS. Functional benefit and convenience factors reflect a strong effect on this variable. To increase the UV in the shopping malls, management may begin the examination of any congestion in the queues to the cashier, congestion in the elevator, or walking path in the mall would be a good start. Mall management should also consider utilizing the mall map guide access that may aid shoppers in their mall shopping activity.

Enhancing HV to increase SS does not include the effect of entertainment facilities in our study due to the COVID-19 pandemic. Instead, HV in shopping malls is perceived in terms of the positive and serene feelings from the ambiance and the entirety of the mall and social experience. In order to increase HV, mall management may focus more on making the interior and exterior of the mall visually appealing that will help trigger fun and relaxing emotions from shoppers like the inclusion of appropriate background music.

The government may apply practical rules in the shopping malls such as social distancing and strict sanitation to complement the continuous business of shopping malls. The feel, excitement, and happiness of mallgoers could be capitalized on for economic recovery. By having more customers purchasing products, dining out, and travelling, the recovery from economic loss may be uplifted due to the expense customers are willing to spend. In addition, to promote safety protocols, rules and regulations with regard to cleanliness and new service applying consideration of the COVID-19 pandemic may be applied to reduce virus spread and promote safety.

### 5.4 Limitation of the study

Limitations are still present despite the strong findings and suggestions. First, our study used SERVQUAL five dimensions model by Parasuraman et al. (1988) and analyzed the data through SEM. We assumed that the study may provide a different insight and outcome when different statistical analyses such as data mining are utilized (Ong et al., 2021c; Kliestik et al., 2022). Additionally, studies that relate to factors about shopping mall such as well-being and mall equity may be considered. It is also recommended for future studies to incorporate the mental and emotional effects of the new normal. Moreover, customer preference may also be determined by utilizing a conjoint analysis approach. Second, this study considered a convenience sampling approach for data collection. Though the results presented no bias and were normally distributed, other approaches may be considered for the data collection process. Third, future studies could look at longitudinal studies to firmly establish the conclusions and generalizations on the Behavior Intentions (BI) and its associated variables of Hedonic Value (HV), Utilitarian Value (UV) and Shoppers' Satisfaction (SS). Lastly, future studies could also consider mixed methods to be able to explain the underlying factors of the BI resulting from the HV, UV, and SS.

## 6 Conclusion

Shopping malls are subjected to a lot of changes that affect their overall operations in catering their products and services to customers set by the new normal. The aim of this study was to evaluate whether aspects of service quality and the shopping mall values in shopping malls affect their overall satisfaction and behavioral intention. Considering the 519 respondents analyzed by SEM, our findings showed that tangibles, empathy, and assurance have an influence over shopper's shopping mall satisfaction. Shoppers' values even during a pandemic are also shown to have a strong influence over satisfaction; while convenience and social experience related to the shopping mall values still resulted in a positive effect. Shopper's satisfaction with both shopping mall service qualities and values are found on reflect strongly to their behavioral intention.

Overall, shoppers perceived both service quality and shopping mall values as important aspects of their satisfaction with shopping malls in the Philippines. These positively impacted their behavioral intention even during the COVID-19 pandemic. This finding may be of great use to mall management or any retail service business to consider in their strategic operations to maintain the business amidst the COVID-19 pandemic.

## Declarations

### Author contribution statement

Ardvin Kester S. Ong, Ph.D; Yogi Tri Prasetyo, Ph.D; Barbara Eliza Vallespin: Conceived and designed the experiments; Performed the experiments; Analyzed and interpreted the data; Contributed reagents, materials, analysis tools or data; Wrote the paper.

Satria Fadil Persada, Ph.D; Reny Nadlifatin, Ph.D: Contributed reagents, materials, analysis tools or data; Wrote the paper.

### Funding statement

This research did not receive any specific grant from funding agencies in the public, commercial, or not-for-profit sectors.

## Data availability statement

Data will be made available on request.

### Declaration of interest's statement

The authors declare no competing interests.

### Additional information

No additional information is available for this paper.
